# The impact of smoking on meniscus surgery: a systematic review

**DOI:** 10.1530/EOR-2024-0097

**Published:** 2025-04-01

**Authors:** Jan Zabrzyński, Jakub Pękala, Maria Zabrzyńska, Przemysław Pękala, Łukasz Łapaj, Robert F LaPrade

**Affiliations:** ^1^ Department of Orthopaedics and Traumatology, Faculty of Medicine,Collegium Medicum in Bydgoszcz, Nicolaus Copernicus University in Toruń, Bydgoszcz, Poland; ^2^ International Evidence-Based Anatomy Working Group, Department of Anatomy, Jagiellonian University Medical College, Krakow, Poland; ^3^ Department of General Orthopaedics, Musculoskeletal Oncology and Trauma Surgery, University of Medical Sciences, Poznan, Poland; ^4^ Twin Cities Orthopedics, Edina, Minnesota, USA

**Keywords:** meniscus, smoking, tobacco, knee, meniscus allograft, arthroscopy

## Abstract

**Purpose:**

- To provide a comprehensive, systematic review on the relationship and effects of smoking on clinical outcomes after meniscus surgery.

**Methods:**

- The following combination of keywords was entered into the electronic search engines: meniscus, meniscus repairs, meniscectomy, meniscal tear, meniscus excision AND (smoke OR smoking OR nicotine OR tobacco). The year of the study, country, type of study, number of subjects, medial/lateral/both menisci, body mass index, smoking status, mean age, gender, follow-up, type/pattern of injury, surgical implications and clinical outcomes were recorded.

**Results:**

- A total of 23 studies published in 2013–2024 were included in the analysis. In ten studies, the meniscus injury was associated with an anterior cruciate ligament (ACL) tear. In four studies, the effect of smoking on meniscal allograft transplantation (MAT) was investigated. The neutral effect of smoking on meniscus surgery was revealed in nine studies, and only one of them focused on isolated meniscus pathology and surgery. The negative effect of smoking on meniscus surgery was shown in ten papers, with four papers focused on isolated meniscus tears and six papers presenting data with concurrent ACL reconstructions.

**Conclusions:**

- This systematic review found that the results regarding the impact of smoking on meniscus repair outcomes were conflicting. Nevertheless, MAT and meniscus repair performed in the presence of concurrent ligamentous injury, both being demanding surgical procedures, require reduction of factors that may contribute to failure. Therefore, cessation of smoking in patients undergoing these procedures is highly advised.

## Introduction

Historically, it was believed that menisci play no important role in knee joint function, and that is why open total meniscectomy was routinely performed (1). As it turned out, either lack of meniscal tissue or meniscal tears irreversibly lead to cartilage lesions and osteoarthritis (OA), which raised their importance and crucial role in the congruence and kinematics of the knee joint as a vital contributor (2). The main aim of joint preservation surgery should be meniscal integrity as an essential step in meniscus surgery. Menisci play an important role in knee joint biomechanics, and in addition to being passive joint stabilizers, they are also involved in facilitating load transmission, shock absorption and proprioception, as well as cartilage nutrition, lubrication and protection. The main goal in joint preservation surgery is to re-establish meniscal tissue integrity in order to fulfill their role in the knee joint: being shock absorber, facilitate load transmission, participating in proprioception, as well as articular cartilage nutrition, lubrication and protection (1, 3, 4, 5, 6).

‘Save the meniscus’ became a slogan of the new era of meniscus surgery. Untreated meniscal tears and meniscectomy were overshadowed by minimally invasive surgery and joint preservation techniques such as meniscal repairs, meniscal allografts and scaffolds. The main purpose was to slow down cartilage degeneration and OA progression (1, 4, 5, 7, 8).

Meniscal tears are the second most common knee joint injury, which is thought to be underestimated (9). The menisci are subjected to various stresses during knee joint motion: tensile, compressive and shear (10). Snoeker *et al.* recognized squatting, kneeling, crawling, chair sitting, stair climbing, lifting and walking as risk factors for meniscal tears (11). Furthermore, the association between increased cartilage loss in the involved compartment and meniscal tears has been reported (12).

The negative impact of smoking on conditions such as neoplasms, endocrine disorders, cardiovascular disease and bone healing has been reported (13). In orthopedic surgery, tobacco use has become a crucial negative healing factor (14). However, the literature regarding the impact of smoking on meniscal surgery is ambiguous. Some authors claim that tobacco use adversely affects results of meniscal surgery, while others did not find such a correlation (15, 16). Thus, the purpose of this study was to provide a comprehensive, systematic review on the relationship and effects of smoking on clinical outcomes after meniscus repair surgery. The investigation hypothesized that the known negative effects of nicotine, such as vasoconstriction and hypoperfusion, may affect meniscal tissue healing (14, 17).

## Materials and methods

### Search strategy

**Figure 1:**
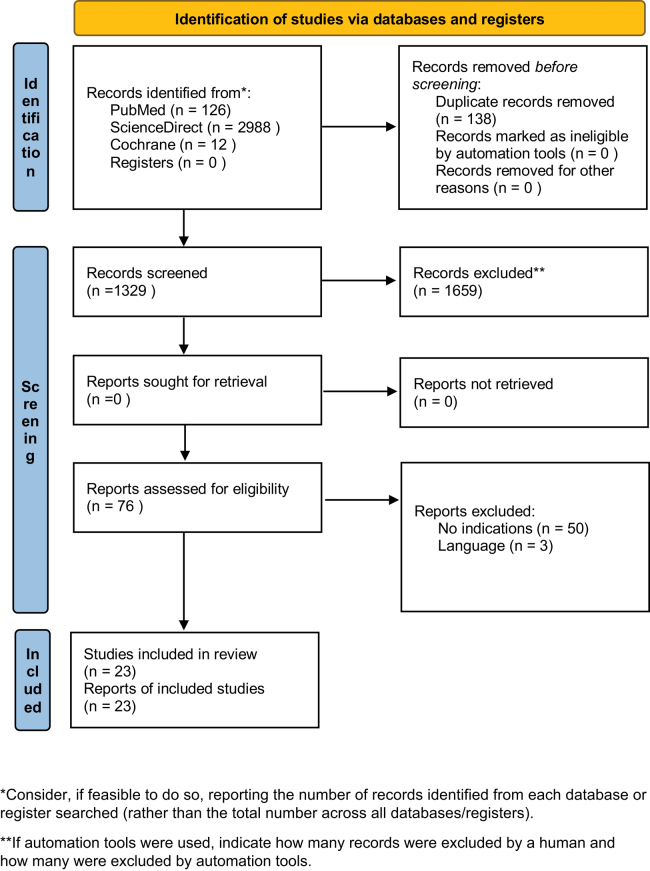
PRISMA 2020 flow diagram for new systematic reviews, which included searches of databases and registers only. *Consider, if feasible to do so, reporting the number of records identified from each database or register searched (rather than the total number across all databases/registers). **If automation tools were used, indicate how many records were excluded by a human and how many were excluded by automation tools.

To identify all of the essential studies that reported relevant information and data concerning the association of meniscus surgery and smoking, an extensive search of the major and significant electronic databases (PubMed, Cochrane Central, ScienceDirect) was performed by two independent authors (JZ, JP). A systematic investigation was conducted from August 2022 to 2024, using combinations of the following key terms:(meniscus OR meniscus repairs OR meniscectomy OR meniscal tear OR meniscus excision) AND (smoke OR smoking OR nicotine OR tobacco)with no limits regarding the year of publication. Moreover, an additional intensive search through the references of all identified studies was conducted. A systematic review of the collected literature was carried out according to the guidelines of Preferred Reporting Items for Systematic Reviews and Meta-Analyses (PRISMA) (Supplementary material (see section on Supplementary materials given at the end of the article)). The PRISMA checklist of our project was presented in Fig. 1. The study was submitted to PROSPERO (International prospective register of systematic reviews).

### Eligibility assessment

Screening of databases was carried out independently by two authors (JZ, PP). After the database search, three independent reviewers (JZ, JP, MZ) screened all the papers and identified a title, abstract and full text concerning smoking and clinical outcomes after meniscus surgery, also with the presence of concurrent ligament and cartilage lesions in the knee. Clinical human studies, levels of evidence I–IV, in the English language, were evaluated and analyzed in this systematic review. Non-English language studies, case studies, reviews, letters to editors, conference abstracts or studies containing incomplete or irrelevant data were not eligible for inclusion (level of evidence V). Meniscal surgery was defined as meniscal repair, allograft transplantation and partial meniscectomy/debridement. Exclusion criteria were as follows: any clinical outcomes and basic science studies analyzing outcomes after surgery in any joint other than the knee. Papers without clearly described outcomes after surgical treatment of the meniscus were excluded. The senior author and expert in evidence-based medicine (RL) made the final decision in case of disagreement among the authors.

### Data extraction

Three independent reviewers (JZ, JP, MZ) extracted the initially screened and relevant data, including the year of the study, country, type of the study, number of subjects, medial/lateral/both menisci, body mass index (BMI), smoking status, mean age, gender, follow-up, type/pattern of injury, surgical implications and clinical outcomes.

### Risk of bias assessment

The risk of bias assessment was performed using the Cochrane collaboration’s risk of bias tool. Risk of bias was assigned to the following domains as ‘low’, ‘high’ or ‘unclear’: sequence generation/allocation concealment (selection bias), blinding of participants and personnel (performance bias), blinding of outcome assessment (detection bias), incomplete outcome data (attrition bias), selective outcome reporting (reporting bias) and other sources of bias. The quality of papers was assessed independently by two reviewers, with agreement.

## Results

A total of 22 studies published in between 2013 and 2024 were included in the analysis. The general characteristics and demographic data were presented in Table 1. Most of the studies were level IV evidence (*n* = 15) and level III evidence (*n* = 5). There was one study with level I evidence and one with level II evidence. In 20 papers the authors investigated the medial meniscus, in 15 papers both the medial and lateral meniscus and for two studies specific data about meniscus laterality were not presented. The total number of included subjects was *n* = 120,646 from which *n* = 19,648(16.3%) were smokers. However, in the study by Laurendo *et al.* the exact number of smokers was not mentioned. There were *n* = 66,405 males and *n* = 54,225 females, although Basques *et al.* did not report the exact number of the male/female population, having presented the percentage instead. Recalculation of absolute numbers was required in that case. The mean age of the subjects was 36.6 years. Only five studies specifically explored the effects of smoking on isolated meniscal tears (Supplementary Table 1). In 10 studies the meniscus injury was associated with an ACL tear and chondral pathology (2) and OA (1). The follow-up duration was presented in 19 studies, and the mean follow-up was 4.9 months (range, 1.0–81.6) (Supplementary Table 1 and Table 2). The surgical method of treatment was defined in six studies and mainly it was an all-inside repair or a meniscectomy. BMI was indicated in 14 studies with a mean value of 30.4. In four studies the effect of smoking on meniscal allograft transplantation (MAT) was investigated (Table 2). Moreover, there were two multicenter cohort studies on ACL injuries with concomitant meniscal repair surgeries (Supplementary Table 1).

**Table 1:** The general characteristics and demographic data of the included studies on meniscus surgery and smoking.

Reference	Study type	LOE	Country	Publication year	Meniscus	Subjects, *n*	Smokers	Male/female	Mean age (years)
Blackwell *et al.* (18)		III	USA	2016	Medial meniscus 65%; lateral meniscus 27%; both menisci 8%	104	52	32/20	27.9
Johnsen *et al.* (19)	CSS	IV	Denmark	2019	n/a	620	138	80/54	45
Moses *et al.* (15)	RCS	IV	USA	2017	Medial and lateral meniscus	51	10	34/17	25
Cox *et al.* (20)	PCS	I	USA	2014	Medial and lateral meniscus	1411	135	785/626	23
Buyukkuscu *et al.* (21)	RCS	IV	Turkey	2019	Medial meniscus	33	15	23/10	46.1
MOON knee group *et al.* (22)	COS	II	USA	2020	Medial and lateral meniscus	3276	302	1433/1843	23
Uzun *et al.* (23)		IV	Turkey	2019	Lateral meniscus	43	12	37/6	29.5
Laurendon *et al.* (24)	RCS	IV	France	2017	Medial meniscus 58.6%; lateral meniscus 36.8%; both menisci 4.6%	87	n/a	61/26	28.5
Astur *et al.* (25)	PCS	III	Brazil	2018	107 isolated ACL tears; 72 ACL tears + medial meniscus injury; 60 isolated medial meniscus tears	239	51	196/43	33
Uzun *et al.* (26)	CS	IV	Turkey	2017	Medial meniscus	80	30	76/4	29.1
Basques *et al.* (27)	CCS	III	USA	2015	n/a	17774	15.7%	53.1%/46.9%	53
Haviv *et al.* (28)		IV	Israel	2015	Medial and lateral meniscus	201	59	133/68	44.4
Haklar *et al.* (29)	CS	IV	Turkey	2013	Medial meniscus	112	46	94/18	34.57
Antosh *et al.* (30)	TS	IV	USA	2019	Medial and lateral meniscus	104	40	95/9	28
Waterman *et al.* (31)	CS	IV	USA	2016	Medial and lateral meniscus	230	84	203/24	27.2
Beletsky *et al.* (32)	TCS	IV	USA	2020	Medial and lateral meniscus	126	17	72	48.9
Franovic *et al.* (33)	CS	IV	USA	2020	Medial and lateral meniscus	166	20	85/81	55.2
Domżalski *et al.* (16)	RCS	IV	Poland	2021	Medial and lateral meniscus	92	47	36/56	31.5
Zaffagnini *et al.* (34)	CS	IV	Italy	2016	Medial and lateral meniscus	147	18	117/30	44.8
Heyer *et al.* (35)	CS	IV	USA	2019	Medial and lateral meniscus	95,191	15,781	53,138/42,053	47.1
Zabrzyński *et al.* (36)	CS	III	Poland	2022	Medial meniscus	50	17	32/18	41.68
Kraus *et al.* (37)	RCPS	III	USA	2021	Medial and lateral meniscus	509	39	206/301	n/a

CS, case series; CCS, case-control study; COS, cohort study; CSS, cross-sectional study; LOE, level of evidence; PCS, prospective cohort study; RCS, retrospective cohort study; RCPS, retrospective comparative study; TS, therapeutic study; TCS, therapeutic case series.

**Table 2:** Smoking as a risk factor in meniscal allograft transplantation surgery.

Reference	Mean follow-up	BMI	Type of injury	Surgical implications	Outcomes	Overall effect
Antosh *et al.* (30)	n/a	n/a	n/a	Meniscal allograft transplantation	Return to duty at more than 2 years (OR = 0.48 (95% CI: 0.13–1.74; *P* = 0.267); there was no difference in return-to-duty rates between tobacco smokers and non-tobacco users	Neutral
Zaffagnini *et al.* (34)	4 years	25.2	n/a	Meniscal allograft transplantation	There was no difference reported between tobacco smokers and non-tobacco users	Neutral
Waterman *et al.* (31)	n/a	n/a	n/a	Meniscal allograft transplantation	Tobacco use was associated with significantly increased risk of failure (OR = 2.22; 95% CI: 1.19–4.17; *P* = 0.028)	Negative

BMI, body mass index.

### Neutral effect of smoking on meniscus surgery

This section included nine studies. Only Zabrzyński *et al.* focused on isolated meniscus pathology and surgery (18). The follow-up time ranged from 3 to 72 months. The mean BMI was 25.88. In three studies meniscal lesions were associated with OA; Johnsen *et al.* reported the prevalence of OA in 37.7% of smokers, Buykkuscu *et al.* in >90%, while Beletsky *et al.* in 70.6% (36, 37, 38). No significant relationship between smoking and OA was found. Furthermore, in the study by Buyukkuscu *et al.* no difference between smoker and non-smoker groups (*P* > 0.05) was shown regarding preoperative and postoperative Lysholm and IKDC scores. Zabrzyński *et al.* also noted no association between smoking indices and functional outcomes after all-inside repair of chronic medial meniscus tear (18). In four studies with concurrent cruciate ligament tears: Moses *et al.* revealed concomitant anterior cruciate ligament (ACL) tears in 54.9% study population, Laurendon *et al.* in 70%, Astur *et al.* in 179/239 and Haviv *et al.* in 11% (15, 19, 20, 21). In the mentioned studies, no association between smoking and impaired clinical results after surgery was found. Laurendon *et al.* and Moses *et al.* reported no link between meniscus postsurgical failure and smoking habit (15, 21). The degenerative character of the injuries predominated; Johnsen *et al.* revealed 50% degenerative injuries in smokers and 59.3% in non-smokers, Buyukkuscu *et al.* presented chronic tears (>8 weeks) in more than 90% of patients, Haviv *et al.* in 55% of their study population and Beletsky *et al.* in 41.27% (20, 22, 23, 24). Buyukkuscu *et al.* reported on the zones of meniscal lesions and 42.4% injuries were in the red-red zone and 57.6% in the red-white zone (23). The medial meniscus was more frequently torn, as shown by Moses *et al.* 74.5%, Buyukkuscu *et al.*>90% and Laurendon *et al.* 58.6% (15, 21, 23). On the other hand, the MOON Knee Group investigated the influence of smoking on clinical outcomes after ACL reconstruction (25). They identified failure risk factors in patients undergoing ACL surgery with concomitant meniscus repair. They concluded that patients who had quit smoking (compared with non-smokers) had a greater risk of subsequent meniscal surgery (25).

### Negative effect of smoking on meniscus surgery

Ten papers were included in this section, although only four papers focused on isolated meniscus tears and six papers presented data with concurrent ACL tears. The follow-up duration ranged from 1 to 72 months. The mean BMI was 25.4. The period between trauma and surgery was defined in three studies and ranged from 29.6 to 122.62 days. In two studies, the surgical technique was defined as an all-inside repair. The failure risk of meniscus repairs in smokers was 27% (*P* = 0.0076) in Blackwell *et al.*, 25% (*P* < 0.05) in Uzun *et al.* and 37.5% (*P* = 0.008) also in Uzun *et al.* study (26, 27, 28). Blackwell *et al.* summarized that the failure risk after meniscus repair was 3.8 times higher for smokers compared to non-smokers (26). In the studies by Uzun *et al.* failures of meniscal repairs occurred mainly in the red-white zones, and failure rates were higher for smokers than for nonsmokers (*P* = 0.008) (27, 28). At the 3-month follow-up after meniscus surgery, Franovic *et al.* reported that ‘never smokers’ experienced significantly greater improvements than ‘former smokers’ or ‘current smokers’ (*P* < 0.048, *P* < 0.035) (29). Some authors suggested that smoking history may prove useful in postoperative outcome prediction. In the study by Haklar *et al.* no association between impaired meniscus healing and smoking, suture type and the length of tear in isolated meniscal injuries (*P* = 0.005) was observed. However, in the group of meniscus tears with a concurrent ACL tear, smoking significantly affected meniscal healing (*P* = 0.05) (30). Moreover, Cox *et al.* performed meniscus surgery along with ACL reconstruction (31). The study population consisted of patients with primary ACL tears and concomitant articular cartilage lesions and meniscal tears (medial-38%, lateral-46%). The authors found that significant predictors of lower outcome scores were lower baseline scores, higher BMI, lower education level, smoking and ACL revisions. Specifically, current smokers and previous smokers had lower IKDC and KOOS scores (31).

### Negative effect of smoking on meniscectomy

Basques *et al.* reported that in 17,774 patients who underwent meniscectomy, smokers had higher odds of readmission (OR: 1.67; *P* = 0.033) (32). Such a correlation was also observed in patients with diabetes and pulmonary diseases. Interestingly, in contrary to smoking, age >65 years did not increase the odds of any studied adverse events. Moreover, Kraus *et al.* observed that patients who failed nonoperative treatments, including injections and physical therapy and also were active smokers at the time of partial meniscectomy had significantly worse baseline and postoperative patient-reported outcome measures (PROMs) compared with nonsmokers (33).

### Smoking and meniscal allograft transplantation

The influence of smoking on clinical outcomes after MAT was investigated by authors of four studies included in this review. Antosh *et al.* reported a high rate of tobacco use in a population that underwent MAT (39%), yet no differences in a military population between tobacco smokers and non-smokers were observed (OR: 0.48 (0.13–1.74); *P* = 0.267). Moreover, no correlation between return to full duty and variables such as age, gender, tobacco use or BMI was reported (34). In addition, Zaffagnini *et al.* did not report any significant differences between both groups (35). On the contrary, Waterman *et al.* with a similarly high rate of smoking among participants (37%), reported a link between tobacco use and increased risk of failure (OR: 2.22; 95% CI: 1.19–4.17; *P* = 0.028) (36).

## Discussion

The most important finding of this systematic review was that smoking may have a major negative impact on meniscus surgery. However, results regarding the impact of smoking on meniscus repair outcomes were conflicting (14, 26, 37, 38, 39, 40, 41, 42, 43, 44).

The most important determinants of successful meniscus surgery are vascularization pattern, nutritional status and morphological pattern of the tear (5). Meniscus blood supply determines the healing potential of a torn meniscus. Medial and lateral genicular arteries constitute a major source of blood supply, but in the mature skeleton, vascularization patterns differ. The red-red zone, which is 10–25% of the meniscus, is supplied by the vessels. Quite the opposite, the white zone, which is nourished by diffusion form the synovial fluid, which forms the inner 1/3 of the meniscus. Between them occurs the red-white zone, with combined features of both. Although newer studies reported that the degree of vascular penetration into the periphery of the lateral and medial meniscus ranges from 0 to 48% and 0 to 42%, respectively (45).

It was believed that meniscal tears can be repaired only in the peripheral zone, but Barber-Westin *et al.* presented meniscus healing potential in the red-white zone with decreased vascularity, with an 86% rate of healed meniscal tears (46). Moreover, it was shown that meniscal repair significantly improved patients’ symptoms at 2 years follow-up regardless of the tear zone (47). In addition, animal studies have revealed that this fibrocartilaginous tissue is able to heal without significant vascular contribution (47).

Seven of the studies included in this review showed no correlation between smoking and postoperative failure. Moses *et al.* suggested that this fact may be associated with the meniscus vascularization pattern and possible vascular effects of smoking (15). Systemic effects of smoking through the circulatory system have been widely studied (48).

Although it is well known that smoking has adverse effects on fracture union, tendon and wound healing, bone mineral density and clinical outcomes of knee ligament surgery, the deleterious effects of smoking on meniscus surgery have not been well defined. In their systematic review, Kanneganti *et al.* in 2014 clearly showed that the current literature highlights the negative effects of smoking on knee ligament and articular cartilage surgery; however, the scarcity of data on meniscus surgery was underlined (14).

We believe that the adverse effects of tobacco use, such as vasoconstriction, hypoperfusion and free radical formation, may have a significant negative impact on meniscus healing (38). Nicotine stimulates the neovascularization process, which is described as ‘pathological’, attributing to newly formed capillaries with abnormal permeability (49). Having adverse effects on microcirculation and promoting pathological angiogenesis, smoking may contribute to the high failure rates following meniscal repairs, which were reported in some of the studies included in this review.

The tissue-specific effects of smoking on the musculoskeletal system are an ongoing subject. Zabrzynski *et al.* in their study, observed a negative correlation between smoking history and the extent of neovascularization and reported that smoking impairs the vascularization of tendons (17). Novikov *et al.* observed that smoking cigarettes is associated with worse objective and subjective outcome measures, as well as increased rates of complications following primary anterior cruciate ligament reconstruction (50). Michaud *et al.* demonstrated the negative effect of smoking on neovascularization and its association with a significant reduction of capillary density in ischemic muscles (51). Smoking has a significant impact on cartilage, resulting in tissue damage and the development of inflammatory processes, which result in an enhanced risk of OA (52).

Kraus *et al.* noted that patients who were active smokers at the time of partial meniscectomy had significantly worse baseline and postoperative PROMs compared with nonsmokers (33). It seems to be clear that tobacco smoking impairs the regeneration of musculoskeletal tissues and subsequently the functional outcomes, although data from our study do not entirely support this statement (11, 18, 23).

The success of meniscus repair surgery relies on several variables. As we presented, the majority of the studies focus on complex injuries of the knee joint, predominantly with associated chondropathy and ACL tears. Interestingly, it was reported that meniscal repairs performed at the time of ACL reconstruction had better outcomes than isolated repairs. However, it was shown that with the use of biological augmentation, such as marrow venting, similar results may be achieved even in the treatment of isolated meniscal lesions (53).

In addition, the morphology of tears may be described as simple or complex, localized in various zones of vascularization. Furthermore, they may be acute or chronic, with the development of degenerative tears. Those so-called ‘degenerative’ tears could be resistant to the influence of smoking, maybe due to already impaired vascularization (18, 54). The issue of higher BMI is also important in meniscus surgery because these patients usually require intervention more frequently. Patients with moderate or severe obesity (BMI >25) have inferior short-term outcomes compared with non-obese patients after partial meniscectomy (54). The association between smoking and worse postoperative functional results in ACL reconstructions, as well as delayed bone healing and wound healing capacity after this surgery, is well known (48, 49). The negative impact of smoking was predominantly observed in complex knee injuries with concurrent ACL tears. Interestingly, no significant impact of tobacco use on clinical outcomes has been proven in the population with OA (24). Moreover, in several studies with a concurrent ACL tear, a neutral association between clinical outcomes and smoking was presented (15, 19, 20, 21, 28). Two high-level evidence, multicenter, cohort studies reporting results regarding risk factors for unsuccessful ACL reconstruction with concomitant meniscus tears were included in this review. Cox *et al.* concluded that smokers and ex-smokers had lower postoperative functional scores (31). Although the MOON Knee Group results showed an overall neutral effect of smoking on meniscus surgery, they stated that ex-smokers had a greater likelihood of having subsequent meniscus surgery (25). In light of this review, in patients undergoing knee ligament surgery with concomitant meniscus repair, cessation of smoking is a rational recommendation. This might reduce the risk of postoperative meniscus failure, especially in full-thickness tears with vertical, longitudinal and bucket-handle patterns in the red-white zone with limited vascularization (28).

Subsequent meniscal surgery can have psychological, economic and social effects on patients and also on the national health system (55, 56). Cancienne *et al.* observed higher rates of reoperation, infection and venous thromboembolism (VTE) in smokers undergoing ACL reconstruction (57). Considering the high percentage of meniscal tears in patients undergoing revision ACL reconstruction, eliminating modifiable risk factors such as smoking for these types of surgeries should lower the failure rates, which ultimately reduces the economic burden on the national health system (58).

Irreparable meniscal tears treated with subtotal or total meniscectomy ultimately lead to a predictable pattern of progressive joint degeneration, particularly within the lateral compartment (36). MAT is a treatment modality for patients with persistent or recurrent symptoms after partial meniscectomy and a remnant meniscus (34). Numerous authors recommend MAT for physically active patients with meniscus insufficiency to diminish pain and, potentially, prevent or slow down the development of OA (59, 60).

Lee *et al.* noted that additional procedures (such as realignment osteotomy, ligament reconstruction and articular cartilage repair) are performed simultaneously or in a staged manner along with MAT in more than 50% of total MATs (61).

However, the failure rate after MAT may be discouraging, ranging from 0% up to 87.5% (62, 63, 64, 65). There has been discussion on the exact risk factors of MAT surgery failure. We presented two studies that focused on tobacco use in patients who underwent MAT. These papers presented contradictory results. Nevertheless, MAT is a very demanding surgical procedure dedicated for high-volume surgeons and potential candidates should minimize their potential risk factors, including cessation of smoking.

The recent literature contains reviews about meniscal repair and failure risks. In a systematic review, Hamilton *et al.* did not find a significant difference between male and female patients (66). Rothermel *et al.* revealed no significant difference in the meniscus repair failure rate between patients older than 40 and younger than 40 (67). In the review by Everhart *et al.* factors such as the chronicity of the tear, surrounding cartilage health and age showed no significant difference in failure rates in patients over 40 years of age who underwent meniscal repair (68). A 5-year follow-up meta-analysis of meniscus repair was published in 2012 by Nepple *et al.* (69). They found an increased rate of failure over time, especially after 2 years of repair.

There were some limitations of this systematic review. First, the methodology of the included studies, the concurrent lesions of the knee joint (mostly ACL tears and chondral injuries), operative methods (meniscus suturing, partial meniscectomies), follow-up period and study designs varied significantly. Second, the literature on this topic mostly includes retrospective level III and IV evidence studies. Third, a source of selection bias was also inherent, as only English-language studies were included. In addition, the included clinical studies only examined tobacco smoking and not smokeless tobacco or other forms of nicotine uptake. We supported a well-established tool to minimize the risk of bias; however, we realize that there are various scales used for the assessment of paper quality. In the majority of included studies, we observed an overall low risk of bias; however, some studies presented limitations with an unclear risk of bias, such as: random sequence generation in four papers (domain 1), allocation concealment in two papers (domain 1), blinding of participants and personnel in five papers (domain 2), incomplete outcome data in two papers (domain 4), selective reporting in five papers (domain 5) and various bias problems in four papers (domain 6).

## Conclusions

Overall, smoking may have a negative impact on meniscus surgery. However, data regarding the impact of smoking on meniscus repair outcomes were conflicting. Nevertheless, MAT and meniscus repair performed in the presence of concurrent ligamentous injury, both being demanding surgical procedures, require the reduction of factors that may contribute to failure. Therefore, cessation of smoking in patients undergoing these procedures is highly advised.

## Supplementary materials



## ICMJE Statement of Interest

The authors declare that there is no conflict of interest that could be perceived as prejudicing the impartiality of the work.

## Funding Statement

This work did not receive any specific grant from any funding agency in the public, commercial or not-for-profit sector.

## Author contribution statement

JZ and JP contributed to the conceptualization of the study. The methodology was developed by JZ, JP, MZ and PP. JZ and ŁŁ handled the software. JZ was responsible for validation. Formal analysis was performed by JZ, JP and MZ. The investigation was conducted by JZ, JP and MZ. Resources were provided by PP and ŁŁ. JZ, JP and PP curated the data. JZ and JP prepared the original draft. MZ, PP and ŁŁ contributed to writing, review and editing. Visualization was carried out by JZ and JP. ŁŁ and RL supervised the project. Project administration was managed by JZ. All authors have read and agreed to the published version of the manuscript.
