# Supplementary material for: The impact of smoking on meniscus surgery: a systematic review
Source: EFORT Open Rev. 2025 Apr 1;10(4):193–202. doi: 10.1530/EOR-2024-0097 (PMC12002735; doi:10.1530/EOR-2024-0097)
Supplement: Supplementary file 1 [file supplementary_materials.pdf]

Supplementary Table I. The influence of smoking on meniscus surgery and clinical outcomes(studies on meniscus repair surgery marked with asterisk).

| Author | Mean follow-up | BMI | Type of injury | Surgical implications | Outcomes | Overall effect |
|---|---|---|---|---|---|---|
| Johnsen et al. [1] | n/a | 26.7 | 50% of meniscus injuries in smokers were degenerative; 59.3% of meniscus injuries in non-smokers were degenerative | n/a | The prevalence of knee OA in patients with meniscus tear was 37.7% (52 of 138) in current smokers and 45.0% (217 of 482) in non-smokers; there was no statistically significant relationship between current smoking and knee OA | Neutral |
| Moses et al. [2]* | 39.9 months | 25 | 38 medial meniscus ruptures (74.5%),13 lateral meniscus ruptures (25.5%);<br><br>28 of the 51 patients (54.9%) had a concomitant ACL injury | 38 medial meniscus repairs (74.5%),13 lateral meniscus repairs (25.5%); 28 of the 51 patients (54.9%) had a concomitant primary ACL reconstruction (24 patients) and revision ACL reconstruction (4 patients) | There was no association between smoking and failed meniscus repair (p=0.7706) | Neutral |
| Buyukkuscu et al. [3]* | 31.1 months | n/a | Medial meniscus tear and chondropathy in >90% of patients, (chronic tears >8weeks, longitudinal, 1-3 cm)<br><br>Zones: red–red (42.4%) and red–white (57.6%) | n/a | The preoperative and postoperative Lysholm and IKDC scores did not significantly differ in the smoker and non-smoker groups (p>0.05)<br>There was no statistically significant difference between the smokers and non-smokers in terms of the distribution of patients with at least one physical examination finding before and after surgery (p>0.05) | Neutral |

| | | | | | | |
|---|---|---|---|---|---|---|
| Laurendon et al. [4]* | 31 months | n/a | 58.6% of lesions (n=51) concerned the medial meniscus, 36.8% (n=32) the lateral meniscus, and 4.6% (n=4) both<br><br>61% (n=53) involved one segment, 25.3% (n=22) two, and 13.8 % (n=12) three (bucket-handle lesion).<br><br>70% had a concurrent ACLinjury | n/a | Smoking was not associated with failure after all-inside meniscus repair | Neutral |
| Astur et al. [5] | 6 months | n/a | 107 patients had an isolated ACL injury (group 1), 72 an ACL tear associated with the meniscal injury (group 2), and 60 cases had an isolated meniscal injury (group 3). | n/a | 6 months after the surgical treatment, there was no difference in quality of life between the groups assessed | Neutral |
| Haviv et al. [6] | 13.5 months | 26.9 | Meniscus tear patterns: bucket handle 24 (12%), radial 55 (28%), discoid 6 (3%), degenerative 110 (55 %)<br><br>Cruciate ligaments tears 23 (11 %) | Partial meniscectomy | Smoking was not correlated with worse post-operative function | Neutral |

| | | | | | | |
|---|---|---|---|---|---|---|
| Beletsky et al. [7] | 24 months | 25.9 | Meniscus tear patterns: degenerative 52 (41.27%), complex 26 (21.43%), flap20 (15.75%), radial 18 (14.29%), root 11 (8.66%), oblique 6 (4.76%), bucket handle 5 (5.56%), vertical 4 (3.17%), discoid 1 (0.79%)<br><br>Pre-existing arthritis had 89 patients (70.6%) Osteochondral defects had 32 patients (25.4%) | | Important factors for consideration of the timeline of achieving clinically significant outcome doesn't include smoking | Neutral |
| MOON Knee Group et al.[8] | 6 years | 24.9 | ACL tears in all population | Primary ACL repair in 3059 subjects, revision in 217 subjects reconstruction type<br><br>Medial menis-cal repair and/or meniscectomy occurring almost twice as frequently as lateral meniscal repair and/or meniscectomy (7.4% vs 4.3%, respectively) | Patients who had quit smoking (compared with nonsmokers) had greater likelihood of subsequent meniscal surgery | Neutral |

| | | | | | | |
|---|---|---|---|---|---|---|
| Zabrzyński et al. [9]* | 6 months | n/a | 37 patients had simple medial meniscus tear (longitudinal, radial, horizontal) and 13 patients had complex medial meniscus tear | All-inside sutures used in meniscal repairs was 3 (range 2–7; SD = 1.2). | No association was found between smoking indices and functional outcomes after all-inside repair of chronic medial meniscus tear. | Neutral |
| Blackwell et al.[10]* | 13 months | 26.5 | Meniscus injury + ACL tears in 71% of patients | All-inside suture - 37 (71 %),inside-out -13 (25 %), outside-in - 2 (4 %) | 15 failures in 56 menisci; 27 % failure risk (p=0.0076);<br>the risk of meniscus repair failure was 3.8 times higher for smokers compared to non-smokers | Negative |
| Uzun et al. [11]* | 63.2 months | n/a | Lateral meniscus tears; including 22 (51.2%) for vertical longitudinal tears and 21 (48.8%) for bucket-handle tears<br><br>Zones:<br>red-red 23,<br>red-white 20<br><br>ACL tears in 17/43 | All-inside suture in 33, and hybrid method in 10 | Smoking was identified as a risk factor for repair failure.<br>Smokers were found to have a higher rate of repair failure for both vertical longitudinal and bucket-handle repairs (P < .05).<br>Three of these patients (4 failures) were smokers, their meniscal tears were located in the RW zone, and a horizontal or oblique mattress configuration was used in their repairs.<br>the failure rate among smokers was 25.0% (3 failures among 12 repairs) compared with 3.2% for nonsmokers (1 failure among 31 repairs). | Negative |
| Uzun et al. [12]* | 51.2 months | n/a | Bucket handle tears in 67%<br><br>Zones:<br>red-red - 62.5%,<br>red-white - 37.5%<br><br>ACL tears in 56/80 | The mean time between trauma and surgery was 5.6 weeks | Failure rates were higher for smokers than for nonsmokers (9/24, 37.5% vs 3/56, 5.3%; P =0.008).<br>Higher failure rates were detected in red-white zone repairs compared with red-red zone repairs (10/30, 33.3% vs 2/50, 4%)(P=0.004), when repairs were >8 weeks after injury (12/45, 26.6% vs 0/35, 0%)(P=0.008) and in smokers (9/24, 37.5% vs 3/56, 5.3%)(P-0.008) | Negative |
| Basques et al. [13]* | n/a | 17.1 | n/a | Meniscectom y | Smokers have an increased odds of readmission (OR, 1.67;P=0.033) | Negative |
| Haklar et al. [14]* | 48.39 months | n/a | 85/112 of medial | The mean time between trauma and | Among patients with isolated meniscal tears (n =23), the length of tear, smoking status, and suture type | Negative |

| | | | | | | |
|---|---|---|---|---|---|---|
| | | | meniscus tears were 2 cm in length; full thickness tears in 66 (58.9%) cases and partial thickness tears in 46 (41.1%) cases<br><br>ACL tears in 89/112 | surgery was 122.62 days<br><br>Double vertical sutures were used for full-thickness tears, and single vertical sutures were used for partial-thickness tears | had no significant effect on healing (P=0.05)<br><br>Based on univariate analysis including all patients (n =112), concurrent ACL reconstruction at the time of meniscal repair, type of tear (full or partial thickness), length of tear, and smoking significantly affected meniscal healing(P=0.05) | |
| Franovic et al. [15] | 3 months after surgery | 31.5 | n/a | 29.6 days to operation | Smoking status showed significant differences in outcomes scores; "never smokers" displaying significantly greater improvements than "former smokers" or "current smokers" (p<0.048, p<0.035)<br><br>Being a "never smoker" yielded a 13.7 times greater likelihood of achieving good outcomes than those that are "current smoker" | Negative |
| Domżalski et al. [16]* | 24 months | 24.4 | Traumatic isolated meniscus tear | Inside-out technique | Knee Injury and Osteoarthritis Outcome Score (KOOS) was 73 + 15 for the entire population, with significantly higher mean scores for the non-smokers' group (80.2 + 13.6) as compared to the smokers' group (67.4 + 14.7) (p ¼ 0.001) | Negative |
| Heyer et al. [17] | 1 month after surgery | 30.8 | n/a | Knee arthroscopy with meniscectomy (medial or lateral) (KAM), ( knee arthroscopy with meniscectomy (medial and lateral) (KAB), knee arthroscopy with chondroplasty (KAC), knee arthroscopy with anterior cruciate | Smokers have significantly higher odds of having any complication event (the composite outcome) when they underwent knee arthroscopy with meniscectomy of medial and lateral menisci (KAB).<br><br>Smoking did not affect any examined thirty-day complication or mortality for knee arthroscopy with meniscectomy (medial or lateral) (KAM) or knee arthroscopy with chondroplasty (KAC).<br><br>Smoking was associated with increased wound complications following knee arthroscopy with anterior cruciate ligament (KACL) reconstruction, as well as cardiac, sepsis, wound, and pulmonary complications following knee | Negative |

| | | | | | | |
|---|---|---|---|---|---|---|
| | | | | ligament reconstruction (KACL) | arthroscopy with medial and lateral meniscectomy (KAB). | |
| Cox et al. [18] | 2 and 6 years | 22.3 | ACL tears in all population<br><br>Medial meniscal pathology was noted in 38% of subjects (19% of those undergoing partial excisions, 13% undergoing repairs, and 7% undergoing no treatment for their tears) Lateral meniscus pathology was noted in 46% of subjects (28% of the cohort undergoing partial excisions, 6% undergoing repairs, and 18% undergoing no treatment) | Primary ACL repair in 1278 subjects, revision in 122 subjects | Current smokers and previous smokers have lower IKDC and KOOS scores | Negative |
| Kraus et al.[19] | 24 months | n/a | Confirmed meniscal pathology, and acute meniscal tear or chronic/degenerative tears and failed nonoperative treatments including injections and physical therapy. | Partial meniscectomy | Patients who were active smokers at the time of partial meniscectomy had significantly worse baseline and postoperative patient-reported outcome measures (PROMs) compared with nonsmokers. | Negative |


1. Johnsen, M.B.; Pihl, K.; Nissen, N.; Sørensen, R.R.; Jørgensen, U.; Englund, M.; Thorlund, J.B. The Association between Smoking and Knee Osteoarthritis in a Cohort of

Danish Patients Undergoing Knee Arthroscopy. *BMC Musculoskelet Disord* **2019**, *20*, 141, doi:10.1186/s12891-019-2518-z.
2. Moses, M.J.; Wang, D.E.; Weinberg, M.; Strauss, E.J. Clinical Outcomes Following Surgically Repaired Bucket-Handle Meniscus Tears. *The Physician and Sportsmedicine* **2017**, *45*, 329–336, doi:10.1080/00913847.2017.1331688.
3. Buyukkuscu, M.O.; Misir, A.; Hamrayev, A.J.; Ozcafer, R.; Cetinkaya, E. Moon. *J Orthop Surg (Hong Kong)* **2019**, *27*, 230949901983630, doi:10.1177/2309499019836300.
4. Laurendon, L.; Neri, T.; Farizon, F.; Philippot, R. Prognostic Factors for All-inside Meniscal Repair. A 87-Case Series. *Orthopaedics & Traumatology: Surgery & Research* **2017**, *103*, 1017–1020, doi:10.1016/j.otsr.2017.05.025.
5. Astur, D.C.; Sbampato, I.N.; Arliani, G.G.; Franciozi, C.E. da S.; Debieux, P.; Cohen, M. ASSOCIATION OF TOBACCO DEPENDENCE, ALCOHOLISM AND ANABOLIC STEROIDS WITH MENISCOLIGAMENTOUS INJURIES. *Acta ortop. bras.* **2018**, *26*, 236–239, doi:10.1590/1413-785220182604172699.
6. Haviv, B.; Bronak, S.; Kosashvili, Y.; Thein, R. Which Patients Are Less Likely to Improve during the First Year after Arthroscopic Partial Meniscectomy? A Multivariate Analysis of 201 Patients with Prospective Follow-Up. *Knee Surg Sports Traumatol Arthrosc* **2016**, *24*, 1427–1431, doi:10.1007/s00167-015-3590-z.
7. Beletsky, A.; Gowd, A.K.; Liu, J.N.; Manderle, B.J.; Yanke, A.B.; Forsythe, B.; Cole, B.J.; Verma, N. FRANO. *Arthroscopy, Sports Medicine, and Rehabilitation* **2020**, *2*, e723–e733, doi:10.1016/j.asmr.2020.06.002.
8. MOON Knee Group; Sullivan, J.P.; Huston, L.J.; Zajichek, A.; Reinke, E.K.; Andrish, J.T.; Brophy, R.H.; Dunn, W.R.; Flanigan, D.C.; Kaeding, C.C.; et al. Incidence and Predictors of Subsequent Surgery After Anterior Cruciate Ligament Reconstruction: A 6-Year Follow-up Study. *Am J Sports Med* **2020**, *48*, 2418–2428, doi:10.1177/0363546520935867.
9. Zabrzyński, J.; Paczesny, Ł.; Zabrzyńska, A.; Huri, G.; Graboń, K.; Pielak, T.; Kruczyński, J.; Łapaj, Ł. Smoking Has No Influence on Outcomes after Repair of the Medial Meniscus in the Hypo and Avascular Zones—A Pilot Study. *IJERPH* **2022**, *19*, 16127, doi:10.3390/ijerph192316127.
10. Blackwell, R.; Schmitt, L.; Flanigan, D.C.; Magnussen, R.A. John. *Orthopaedic Journal of Sports Medicine* **2014**, *2*, 2325967114S0011, doi:10.1177/2325967114S00115.
11. Uzun, E.; Misir, A.; Kizkapan, T.B.; Ozcamdalli, M.; Akkurt, S.; Guney, A. Evaluation of Midterm Clinical and Radiographic Outcomes of Arthroscopically Repaired Vertical Longitudinal and Bucket-Handle Lateral Meniscal Tears. *Orthopaedic Journal of Sports Medicine* **2019**, *7*, 232596711984320, doi:10.1177/2325967119843203.
12. Uzun, E.; Misir, A.; Kizkapan, T.B.; Ozcamdalli, M.; Akkurt, S.; Guney, A. Factors Affecting the Outcomes of Arthroscopically Repaired Traumatic Vertical Longitudinal Medial Meniscal Tears. *Orthopaedic Journal of Sports Medicine* **2017**, *5*, 232596711771244, doi:10.1177/2325967117712448.
13. Basques, B.A.; Gardner, E.C.; Varthi, A.G.; Fu, M.C.; Bohl, D.D.; Golinvaux, N.S.; Grauer, J.N. Risk Factors for Short-Term Adverse Events and Readmission After Arthroscopic Meniscectomy: Does Age Matter? *Am J Sports Med* **2015**, *43*, 169–175, doi:10.1177/0363546514551923.
14. Haklar, U.; Donmez, F.; Basaran, S.H.; Canbora, M.K. Results of Arthroscopic Repair of Partial- or Full-Thickness Longitudinal Medial Meniscal Tears by Single or Double Vertical Sutures Using the Inside-Out Technique. *Am J Sports Med* **2013**, *41*, 596–602, doi:10.1177/0363546512472046.

15. Franovic, S.; Kuhlmann, N.A.; Pietroski, A.; Schlosser, C.T.; Page, B.; Okoroha, K.R.; Moutzouros, V.; Makhni, E.C. Preoperative Patient-Centric Predictors of Postoperative Outcomes in Patients Undergoing Arthroscopic Meniscectomy. *Arthroscopy: The Journal of Arthroscopic & Related Surgery* **2020**, S0749806320308987, doi:10.1016/j.arthro.2020.10.042.
16. Domzalski, M.; Muszynski, K.; Mostowy, M.; Wojtowicz, J.; Garlinska, A. Smoking Is Associated with Prolonged Time of the Return to Daily and Sport Activities and Decreased Knee Function after Meniscus Repair with Outside-in Technique: Retrospective Cohort Study. *J Orthop Surg (Hong Kong)* **2021**, *29*, 230949902110122, doi:10.1177/23094990211012287.
17. Heyer, J.H.; Perim, D.A.; Amdur, R.L.; Pandarinath, R. Impact of Smoking on Outcomes Following Knee and Shoulder Arthroscopy. *Eur J Orthop Surg Traumatol* **2020**, *30*, 329–336, doi:10.1007/s00590-019-02577-2.
18. Cox, C.L.; Huston, L.J.; Dunn, W.R.; Reinke, E.K.; Nwosu, S.K.; Parker, R.D.; Wright, R.W.; Kaeding, C.C.; Marx, R.G.; Amendola, A.; et al. Are Articular Cartilage Lesions and Meniscus Tears Predictive of IKDC, KOOS, and Marx Activity Level Outcomes After Anterior Cruciate Ligament Reconstruction?: A 6-Year Multicenter Cohort Study. *Am J Sports Med* **2014**, *42*, 1058–1067, doi:10.1177/0363546514525910.
19. Kraus, N.R.; Lowenstein, N.A.; Garvey, K.D.; Matzkin, E.G. Smoking Negatively Effects Patient-Reported Outcomes Following Arthroscopic Partial Meniscectomy. *Arthroscopy, Sports Medicine, and Rehabilitation* **2021**, *3*, e323–e328, doi:10.1016/j.asmr.2020.09.021.
